# Immobilisation for Gartland I Supracondylar Humerus Fractures in Children: A Systematic Review

**DOI:** 10.7759/cureus.96418

**Published:** 2025-11-09

**Authors:** Sofia Bitsios, Dominique Dennis, Ramy Elemam

**Affiliations:** 1 Orthopaedics, Norfolk and Norwich University Hospital, Norwich, GBR; 2 Orthopaedics, Broomfield Hospital, Chelmsford, GBR

**Keywords:** 3d printed orthosis, cast, gartland i, immobilisation, paediatric elbow, splint, waterproof cast, supracondylar humerus fracture

## Abstract

Gartland I supracondylar humerus fractures are non-displaced, stable injuries of the paediatric elbow. Optimal immobilisation aims to control pain and protect alignment while minimising disruption to children and families.

This systematic review aims to review the currently available literature on immobilisation and casting options for Gartland I supracondylar fractures, summarising safety (displacement/complications), pain and functional recovery, satisfaction, and practical considerations.

Across randomised and observational studies, type I supracondylar fractures rarely displace irrespective of immobilisation. Compared with collar-and-cuff alone, above-elbow posterior splints or backslabs reduce pain, analgesia use, and sleep disturbance, and accelerate return to activity. Removable (soft) casts and long-arm splints are non-inferior to rigid long-arm casts for radiographic and functional outcomes, while often improving convenience and parent and patient experience. Newer materials (waterproof or hybrid-mesh liners, biobased polyester, and 3D printed orthoses) further enhance comfort without compromising stability.

In conclusion, for Gartland I supracondylar fractures, a well-applied above-elbow posterior splint or removable long-arm cast for around three weeks is typically sufficient. Collar-and-cuff alone is generally inferior for early symptom control. Innovative techniques and casting materials can be offered where available but may require further research to assess their outcomes. Application technique and education remain critical.

## Introduction and background

Supracondylar fractures of the humerus are the most common elbow fracture in children, accounting for up to 60% of all paediatric elbow fractures and most frequently occurring between the ages of five and seven [1]. These injuries typically result from a fall onto an outstretched hand, with hyperextension at the elbow driving the distal humerus into failure [1]. The Gartland classification stratifies extension-type supracondylar fractures into four sub-groups [2], as observed in Table 1 below.

**Table 1:** Summarising the Gartland classification of paediatric supracondylar humeral fractures Table independently compiled by authors from information in Orthobullets and NCBI StatPearls [1,2] NCBI: National Center for Biotechnology Information.

Gartland subtype	Description
Type I	Non-displaced and stable
Type II	Displaced but with an intact posterior cortex hinge. Can be separated into type IIA (no rotational deformity) or type IIB (rotational deformity) as part of the Wilkins-modified Gartland classification
Type III	Completely displaced with cortical disruption
Type IV	Multidirectional instability (defined more recently with arthroscopic and intraoperative studies)

Gartland type I fractures are inherently stable, with periosteal integrity maintained and minimal risk of displacement under normal physiological loads. These injuries do not typically involve neurovascular compromise, and functional outcomes are almost universally excellent with appropriate conservative treatment [2].

Current UK guidance from the British Orthopaedic Association Standards for Trauma (BOAST) and international guidelines from the American Academy of Orthopaedic Surgeons (AAOS) recommend non-operative management with above-elbow immobilisation at 90° of flexion for approximately three weeks [3,4]. The primary purpose of immobilisation is to control pain, protect the child during daily activity, and provide psychological reassurance to caregivers.

Despite the inherent stability of Gartland I supracondylar fractures, practice varies widely across institutions: some advocate simple collar-and-cuff immobilisation, others apply posterior backslabs or circumferential fibreglass casts, and newer approaches explore removable and waterproof devices. This variability reflects a lack of consensus regarding the balance between the rigidity, comfort, and convenience of immobilisation. Since clinical outcomes are similar across modalities, the decision is often shaped by factors such as pain control, family burden, complication risk, and healthcare resource use.

This review summarises and critically appraises the available literature on immobilisation techniques for Gartland I supracondylar fractures, with an emphasis on safety, patient-centred outcomes, and practical considerations.

## Review

Methods

The present systematic review adheres to the Preferred Reporting Items for Systematic Reviews and Meta-Analyses (PRISMA) guidelines. We performed a search of electronic databases, including PubMed, Embase, and the Cochrane Library, covering the period from January 2000 to September 2025. Keywords included “Gartland I”, “supracondylar fractures”, “conservative management”, “casting material”, and “immobilisation technique,” which were used in combination with relevant Boolean operators (AND, OR) where appropriate. Papers written in, or translated into, English were included. The reference lists of all eligible studies were also screened to identify additional relevant publications.

Inclusion criteria comprised studies assessing immobilisation techniques or materials in the management of Gartland I supracondylar humeral fractures. Where such studies were unavailable, particularly for novel immobilisation methods, the search was broadened to include stable paediatric upper-limb fractures. Eligible study designs included clinical trials, cohort studies, case-control studies, systematic reviews, and meta-analyses. Case reports, conference abstracts, and non-peer-reviewed studies were excluded. Studies not available in English, those assessing immobilisation in lower-limb fractures or unstable fractures (or post-fixation cases), or studies involving non-paediatric populations were also excluded.

**Figure 1:**
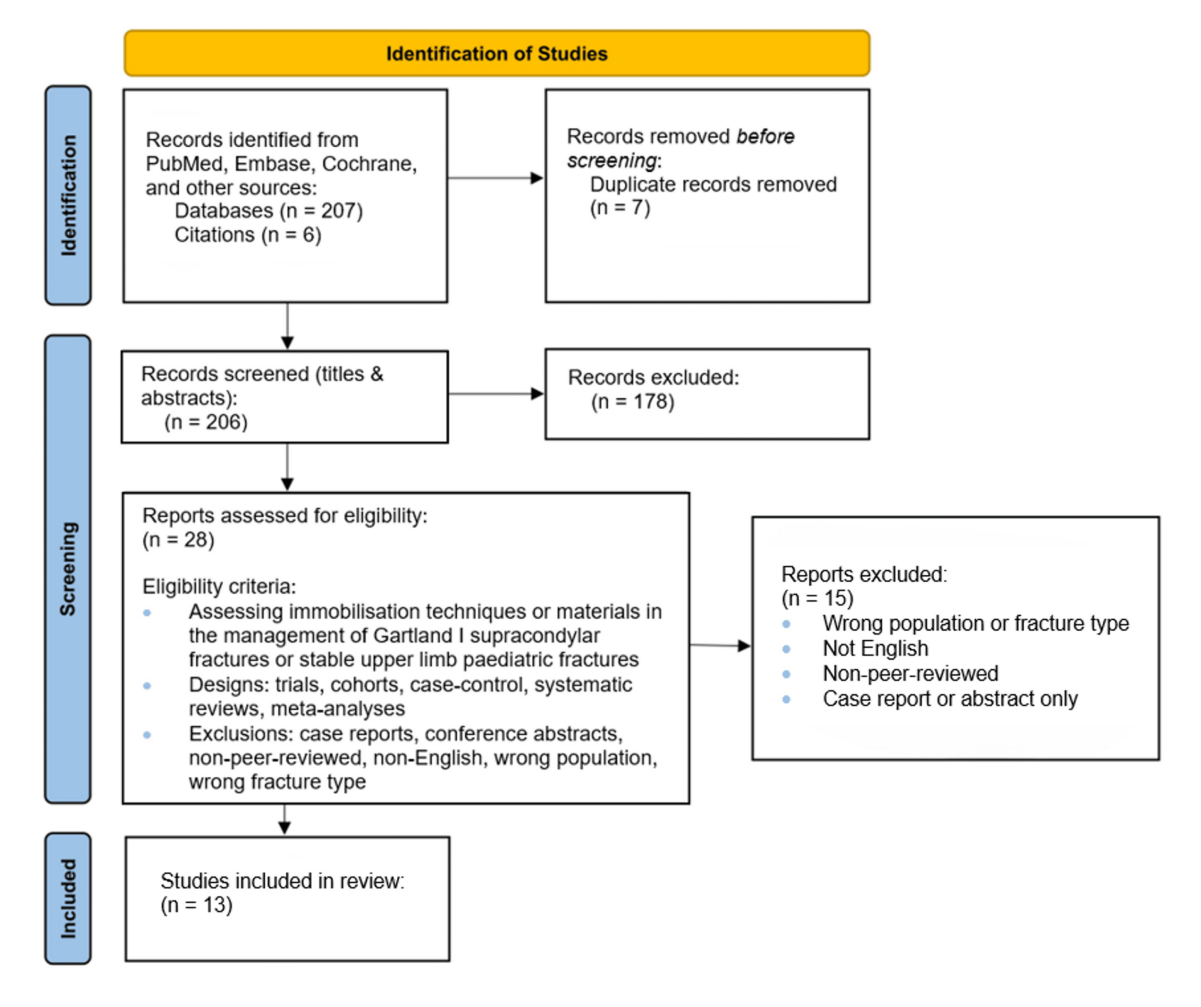
PRISMA diagram outlining screening process for included studies PRISMA: Preferred Reporting Items for Systematic Reviews and Meta-Analyses.

The search yielded a total of 213 papers. Screening of titles and abstracts identified 28 potentially relevant studies, which subsequently underwent full-text review. Three independent reviewers screened all articles, and disagreements were resolved by consensus. About 13 papers met the final inclusion criteria, representing a range of study designs, including randomised controlled trials (RCTs), and providing predominantly Level II-III evidence overall. A PRISMA flow diagram outlining the screening process is presented in Figure 1.

Review

Collar-and-Cuff vs Above-Elbow Backslab

The earliest comparative studies evaluated whether a simple collar-and-cuff sling was sufficient.

Ballal et al. conducted a prospective comparative study of 40 children with type I supracondylar fractures, with non-randomised allocation to either collar-and-cuff (n=20) or above-elbow backslab (n=20). Although both methods prevented displacement, backslabs provided superior pain relief (mean pain score: 3.4 vs 7.2), reduced analgesic use (20% vs 70%), and reduced sleep disturbance (45% vs 85%). Importantly, no fracture displaced in either group, confirming intrinsic stability. The study strongly suggested that collar-and-cuff alone should be avoided due to poor symptom control [5]. However, its conclusions are limited by its small sample size and non-randomised allocation, which may have led to bias.

Oakley et al. conducted a randomised trial of 50 children, comparing backslab plus broad arm sling (n=27) with collar-and-cuff alone (n=23). Results mirrored Ballal: children immobilised in backslabs returned to normal activity faster (median: two days vs seven days) and reported less pain, though the difference in pain scores did not reach statistical significance. Satisfaction rates were similar, and no secondary displacement occurred [6]. While this improved on the evidence base by using randomised groups, the sample size remains small, which therefore means the study may have been underpowered to reach statistical significance in some outcomes (for example, differences in pain scores).

In summary, collar-and-cuff immobilisation maintains fracture stability but is consistently inferior to backslabs in terms of comfort and functional recovery. However, while both studies provided important prospective data, their conclusions may be difficult to extrapolate to larger populations due to lack of long-term follow-up, small sample sizes and therefore insufficient power.

Long-Arm Splints vs Rigid Casts

Cuomo et al. performed a retrospective cohort study of 53 children treated exclusively with posterior long-arm splints. None required conversion to casting or surgery, and no clinically relevant displacement was observed at follow-up. The study concluded that posterior splinting is safe and sufficient for type I supracondylar fractures, challenging the assumption that rigid casting offers any additional benefit [7]. While this study showed clear, clinically relevant outcomes, it is limited by its retrospective design with potential for missing data, small sample size, and short follow-up, which may limit its generalisability.

Siu et al. carried out a randomised trial with an observational extension, comparing removable long-arm splints and above-elbow casts. Though numbers were small (n=15 at six months), outcomes were highly comparable. Radiographic alignment was preserved in both groups, with ≤3° changes in Baumann angle. Functional scores were slightly higher in the splint group, and all splint patients achieved 'excellent' Flynn criteria results. Device issues such as splint breakdown or itchiness were noted but manageable. No significant complications occurred in either group [8]. This was a well-designed RCT with use of validated measures to consider radiographic, functional, and patient-reported outcomes. However, it was limited by its very small final sample size (n=15) and high loss to follow-up, making it underpowered to detect small differences or rare complications. Furthermore, it is important to note that many families declined randomisation.

Coupal et al. synthesised nine studies in a systematic review, concluding that no immobilisation method led to secondary displacement. All modalities, including collar-and-cuff, casts, and splints, produced excellent range of motion outcomes, but splints and removable casts were better tolerated by families and reduced cast-related problems [9]. However, assessment of long-term outcomes were lacking.

Abzug et al. highlighted a caveat: in a prospective evaluation of splints applied in emergency and urgent care settings, 93% had technical flaws and 40% resulted in minor complications such as swelling or skin pressure. This underscores that immobilisation quality, rather than device choice, may determine outcomes in some cases [10].

In summary, posterior splints appear to be effective and are non-inferior to casts. They may even be preferable where convenience and comfort are priorities. However, staff training and application technique are critical to avoid complications.

Rigid Casts vs Removable/Soft Casts

Silva et al. conducted an RCT of 100 children with non-displaced elbow fractures (including Gartland I supracondylar fractures). Participants were treated with either rigid fiberglass long-arm casts or removable soft fiberglass casts. Both groups demonstrated excellent radiographic and functional outcomes with no displacement. Mean range of motion at eight weeks was nearly identical (154°-156°), and satisfaction exceeded 95% in both arms. Parents in the removable cast group were able to remove casts safely at home, avoiding clinic visits. The authors concluded that removable casts provide equivalent fracture protection with additional benefits in convenience, hygiene, and reduced healthcare burden [11]. This study provides more generalisable outcomes, with strong RCT design, adequate sample size, and relevant clinical and radiographic outcomes. However, multicentre RCTs would be required to validate its findings.

Innovations in Casting Materials

Waterproof casts: Silva et al. studied waterproof short-arm casts in stable distal radius fractures. Children had improved early function (+23% in the first two weeks) without added complications, suggesting waterproof technology enhances daily life without compromising safety [12].

Badhe et al. performed a systematic review and meta-analysis of five RCTs (upper limb fractures). Waterproof casts improved comfort, reduced itch, and increased satisfaction, with no increase in complications or unplanned visits [13].

While these studies did not focus on supracondylar fractures, they highlight important developments in casting materials, which could be used to improve patient outcomes in the management of stable supracondylar fractures. Studies focusing specifically on supracondylar fractures would be required to validate these findings and their use in Gartland I supracondylar fractures.

Hybrid-mesh and biobased polyester casts: Ong et al. compared hybrid-mesh casts with conventional fibreglass in 79 children with stable supracondylar fractures (type I/IIa). Both groups maintained fracture stability, but hybrid-mesh casts were lighter, more comfortable, and yielded higher satisfaction scores [14].

Lan et al. randomised 100 children with stable upper limb fractures to fiberglass vs biobased polyester casts. Union was achieved in all cases. Skin problems were significantly fewer with polyester (5 vs 17), and patients rated them superior for comfort, itch, and odour [15].

3D-printed orthoses: Graham et al. conducted a crossover study, finding 3D-printed casts were more comfortable and caused less skin irritation than fibreglass. There were only 12 healthy participants, and each participant only wore each immobilisation method for two hours during testing. However, patients reported enhanced function, comfort, and satisfaction. About 42% of the fibreglass group reported skin irritation [16].

Skibicki et al. tested 3D-printed vs traditional casts in 22 children with acute wrist fractures. Both groups showed >90% maintenance of fracture alignment with no significant difference in skin complications. However, 3D-printed casts offered higher comfort, satisfaction, and ease of care [17].

In summary, novel materials, including waterproof, hybrid-mesh, biobased, and 3D-printed devices, improve patient experience without sacrificing safety. However, these studies remain new with very small sample sizes, and not all studies focused specifically on supracondylar fractures. Therefore, they would require larger prospective, randomised studies to confirm their use in the management of Gartland I supracondylar fractures. Furthermore, novel materials can often be expensive and difficult to obtain, therefore formal evaluation of cost-effectiveness and implementation would be necessary to determine their clinical feasibility.

A summary of all the studies included in the review, including study type, population, primary outcomes, and author recommendations, can be observed in Table 2.

**Table 2:** Summary of all studies included in this systematic review ED: Emergency department; RCT: Randomised controlled trial; ROM: Range of motion; SCH: Supracondylar humerus. Level of evidence: The Oxford Centre for Evidence-Based Medicine hierarchy classifies the strength and quality of studies based on their design. Level I (strongest research): systematic reviews or meta-analyses of randomised controlled trials; Level II: individual randomised controlled trials; Level III: non-randomised comparative or cohort studies; Level IV (weakest research): case series or observational studies without controls [18].

Author (year)	Fracture type	Technique/material	Study design	Sample size (n)	Level of evidence	Primary outcomes	Author's conclusions/recommendations
Ballal et al. (2008) [5]	Gartland I SCH	Collar-and-cuff vs backslab	Prospective comparative	40	II	Backslab ↓ pain, analgesia use, and sleep disturbance; no displacement	Backslab superior for comfort and pain control; collar-and-cuff alone not advised
Oakley et al. (2009) [6]	Gartland I SCH	Backslab + sling vs collar-and-cuff	RCT	50	II	Faster return to activity (two vs seven days); no displacement	Both safe; backslab gives faster recovery and less pain
Cuomo et al. (2012) [7]	Gartland I SCH	Posterior long-arm splint only	Retrospective cohort	53	III	0% conversion or displacement	Posterior splint alone safe and effective definitive management
Coupal et al. (2022) [9]	Gartland I SCH	Multiple immobilisation types	Systematic review	Nine studies (~1,500 pts)	I	No displacement; similar ROM; splints/removable casts ↑ comfort	All methods stable; prefer less restrictive options for comfort/practicality
Silva et al. (2018) [11]	Stable elbow (incl. SCH I)	Rigid vs removable soft cast	RCT	100	II	Equal healing/ROM; high satisfaction; no displacement	Removable casts = rigid casts in safety; improved convenience
Siu et al. (2023) [8]	Gartland I SCH	Long-arm splint vs cast	Pilot RCT + cohort	15 (six-mo follow-up)	II–III	Equivalent alignment and function; minor splint issues	Splints non-inferior to casts; suitable for home management
Abzug et al. (2019) [10]	Mixed paediatric fractures	ED splint quality	Prospective observational	103	III–IV	93% splints flawed; 40% minor skin issues	Application technique crucial; training essential for safety
Silva et al. (2017) [12]	Distal radius (stable)	Waterproof vs standard cast	Randomised crossover	20	II	↑ Physical function (+23% early); no added complications	Waterproof casts improve early function safely
Badhe et al. (2025) [13]	Upper limb (various)	Waterproof vs standard cast	Systematic review and meta-analysis	Five RCTs (~390 pts)	I	↑ Comfort and satisfaction; no ↑ complications	Waterproof casting safe and enhances patient experience
Ong et al. (2024) [14]	SCH I–IIa	Hybrid-mesh vs fiberglass cast	RCT	79	II	Equal stability; lighter, more comfortable cast	Hybrid-mesh casts improve comfort and satisfaction without safety trade-off
Lan et al. (2024) [15]	Stable upper limb	Biobased polyester vs fiberglass cast	RCT	100	II	Fewer skin problems; equal union; ↑ satisfaction	Biobased casts safe, eco-friendly, and better tolerated
Graham et al. (2020) [16]	Healthy volunteers	3D-printed vs fiberglass cast	Prospective crossover	24	III	Better comfort; less irritation; no function loss	3D casts non-inferior to traditional casts; higher comfort
Skibicki et al. (2021) [17]	Stable wrist/forearm	3D-printed vs fiberglass cast	Pilot RCT	22	II–III	Equal healing; ↑ comfort and satisfaction	3D-printed orthoses safe, effective, and well accepted

Discussion

Inherent Stability of Gartland I Factures

A central message across the literature is that Gartland I fractures are structurally stable injuries. They do not displace under physiological loads, as the periosteum and surrounding soft tissues maintain alignment. This stability explains why all studies, whether using collar-and-cuff, posterior splints, rigid casts, or removable devices, report negligible displacement rates. The absence of treatment failure across RCTs and cohorts strengthens the conclusion that immobilisation primarily serves as pain relief and protection, rather than reduction maintenance. From a pathophysiological perspective, these findings highlight that immobilisation should be viewed less as a mechanical necessity and more as a comfort intervention.

Pain Control and Functional Recovery

Pain and activity restriction are the main burdens of type I supracondylar fractures. Both Ballal et al. and Oakley et al. demonstrated clear symptomatic benefits of posterior splints or backslabs over collar-and-cuff alone, with reduced analgesic requirements, improved sleep, and faster return to daily activity [5,6]. Even though the fractures are stable, immobilisation appears to reduce periosteal irritation and muscle spasm, thereby improving comfort. These outcomes matter to families: a quicker return to school, sports, and play is often the key metric of success in a child’s eyes. Thus, immobilisation choice is not trivial, as it directly impacts the child’s quality of life.

Equivalence of Casts and Splints for Healing

Studies such as Silva et al. and Siu et al. demonstrate that splints and removable casts are non-inferior to rigid casts in terms of alignment, radiographic parameters (e.g., Baumann angle and carrying angle), and recovery of range of motion [8,11]. This convergence of findings across multiple study designs and populations underscores that rigid casting offers no stability advantage for type I supracondylar fractures. As such, the decision can focus on patient preference, practicality, and complication profiles, rather than fracture security.

Family-Centred Care and Satisfaction

Parents consistently report greater satisfaction with removable or innovative immobilisation methods. These approaches allow for easier bathing, reduced itching and odour, and avoidance of cast saw removal, a source of anxiety for many children [11-17]. Studies of waterproof and hybrid-mesh liners, biobased materials, and 3D printed orthoses show that families value convenience as much as clinical outcomes [12-17]. This resonates with a broader movement in paediatric orthopaedics toward family-centred care, where treatment success is defined not only by radiographs but also by lived experience and daily functioning.

Complications and Application Quality

While Gartland I fractures themselves are low-risk, complications can arise from the immobilisation method. Abzug et al. reported high rates of technical flaws in splint application, with 40% resulting in soft-tissue or skin issues [10]. These findings reinforce that training and supervision are critical, particularly in emergency settings where immobilisations are often first applied. A well-applied splint can be safe and comfortable, but a poorly applied one can cause preventable harm. This suggests that guideline recommendations should emphasise not only the choice of immobilisation but also standards of application and caregiver education (e.g., warning signs of swelling, skin irritation, or slipping).

Resource Utilisation and Healthcare Implications

Beyond patient outcomes, immobilisation choice affects the healthcare system. Removable casts that can be taken off at home reduce follow-up clinic visits and cast saw use, freeing resources for more complex patients [11]. Waterproof and biobased options may be more expensive upfront, but could reduce unplanned returns due to odour, itching, or skin breakdown [12-15]. 3D printing has potential for customised, lightweight devices, but currently faces logistical and cost barriers [16,17]. In an era of value-based care, these resource considerations are important. Formal cost-effectiveness analyses are still lacking but would be invaluable for informing policy.

International Variability in Practice

Despite consistent evidence, immobilisation practices vary worldwide. Some centres continue to use collar-and-cuff as first-line management, others routinely cast all type I injuries, and many are transitioning to splints or removable casts. These differences reflect local training, traditions, and access to materials. For instance, in resource-limited settings, a simple posterior slab may remain the most feasible option. Conversely, in high-resource environments, families may expect waterproof or custom-moulded solutions. This variability underscores the need for context-specific recommendations, balancing evidence with practicality.

Risk of Bias and Quality Assessment

Overall, the included studies were of moderate-to-high methodological quality, with the majority classified as Level II-III evidence according to the Oxford Centre for Evidence-Based Medicine hierarchy [18]. Among RCTs, most demonstrated low or some concerns about risk of bias using the Cochrane risk of bias 2 (RoB 2) tool [19]. Limitations are typically related to unclear randomisation procedures, small sample sizes, and incomplete reporting of allocation concealment or blinding. Nevertheless, outcome data were generally complete, and objective endpoints such as fracture displacement and range of motion were reliably assessed.

Non-randomised comparative and cohort studies assessed with the Risk of Bias In Non-randomised Studies of Interventions (ROBINS-I) [20] and Newcastle-Ottawa Scale (NOS) [21] generally displayed moderate-to-serious risk of bias, mainly due to confounding (non-random allocation), potential selection bias, and limited adjustment for baseline differences. However, outcome reporting and follow-up were adequate across most studies.

Observational studies and case series (e.g., Abzug et al.) were rated as moderate quality using the JBI checklist [22], with consistent outcome definitions but variable reporting completeness.

The systematic reviews and meta-analyses (Coupal et al. and Badhe et al.) achieved moderate-to-high confidence on AMSTAR 2 [23]. Both conducted duplicate screening and considered risk of bias in synthesis, although one lacked full protocol preregistration.

In summary, while a few older or smaller studies exhibited methodological limitations, the overall evidence base is internally consistent, clinically reliable, and methodologically sound for informing non-operative immobilisation of Gartland I supracondylar fractures. The predominant risks relate to small sample sizes and lack of blinding, rather than fundamental flaws in study conduct. A summary of the design of each study and overall risk of bias and quality assessment can be observed in Table 3.

**Table 3:** Quality assessment of each included study ED: Emergency department; NOS: Newcastle-Ottawa Scale; RCT: Randomised controlled trial; RoB 2: Risk of bias 2; ROBINS-I: Risk of Bias In Non-randomised Studies of Interventions; SCH: Supracondylar humerus.

Study (year)	Design	Assessment tool	Key domains (abridged)	Overall risk/quality	Notes
Oakley et al. (2009) [6]	RCT	RoB 2 [19]	Randomization: some concerns (sequence/concealment not fully described); deviations: low; missing data: low; outcome measurement: low; reporting: low	Some concerns	Non-random allocation; symptom outcomes unblinded
Silva et al. (2018) [11]	RCT	RoB 2 [19]	Randomization: low; deviations: low; missing data: low; outcome measurement: low; reporting: low	Low risk	ED RCT; blinding not feasible; small sample
Siu et al. (2023) [8]	Pilot RCT (with small cohort extension)	RoB 2 (RCT) [19]/ROBINS-I (non-randomised cases) [20]	Randomization: some concerns; missing/attrition: some concerns (small n, loss to follow-up); measurement/reporting: low. Cohort component (ROBINS-I): confounding/selection: moderate; other domains: low-moderate	Some concerns (overall)	No concurrent control; consistent radiographic follow-up
Silva et al. (2017) [12]	Randomised crossover trial	RoB 2 (crossover variant) [19]	Randomisation/period effects: some concerns (small n, potential carryover); deviations: low; missing/measurement/reporting: low	Some concerns	Clear synthesis; heterogeneity; mostly small studies
Ong et al. (2024) [14]	RCT	RoB 2 [19]	Randomization: low; deviations: low; missing data: low; outcome measurement: low; reporting: low	Low risk	Descriptive audit of splint quality; not SCH-specific
Lan et al. (2024) [15]	RCT	RoB 2 [19]	Randomization: low; deviations: low; missing data: low; outcome measurement: low; reporting: low	Low risk	Large single-centre RCT; pragmatic outcomes
Skibicki et al. (2021) [17]	Pilot RCT	RoB 2 [19]	Randomization: some concerns (pilot size, limited detail); deviations: low; missing data: low; measurement/reporting: low	Some concerns	Small, underpowered; notable attrition/device issues
Ballal et al. (2008) [5]	Prospective comparative (non-randomised)	ROBINS-I [20]	Confounding: serious (non-random allocation); selection: moderate; classification of interventions: low; deviations: low; missing data: low; outcome measurement: low–moderate; reporting: some concerns	Serious risk of bias	Distal radius model; function advantage early
Cuomo et al. (2012) [7]	Retrospective cohort	ROBINS-I [20]/NOS [21]	Confounding/selection: serious (no control, retrospective); missing data: some concerns; measurement/reporting: low–moderate	Serious risk (ROBINS-I)/NOS: fair	Small trials; heterogeneity across outcomes
Abzug et al. (2019) [10]	Prospective observational (no control)	JBI case series checklist [22]	Clear criteria, consecutive inclusion: some concerns; complete inclusion/follow-up: some concerns; outcomes clearly reported: yes	Moderate quality	Comfort/satisfaction subjective; stability equivalent
Graham et al. (2020) [16]	Prospective crossover in volunteers	RoB 2 (crossover variant) [19]	Randomization/period effects: some concerns; applicability to patients: limited (volunteers); other domains: low	Some concerns	Patient-reported comfort advantages; equal union
Coupal et al. (2022) [9]	Systematic review	AMSTAR 2 [23]	Protocol preregistration: partial; study selection/duplication: yes; risk of bias accounted for: partial; meta-analysis methods: N/A/appropriate; publication bias: unclear	Moderate confidence	Healthy volunteers; generalisability to fractures limited
Badhe et al. (2025) [13]	Systematic review and meta-analysis	AMSTAR 2 [23]	Protocol: yes; duplicate screening/extraction: yes; RoB incorporated into synthesis: yes; meta-analytic methods: appropriate; publication bias explored: yes	High confidence	Small pilot; comfort/satisfaction favour 3D casts.

Limitations

This systematic review has several important limitations. The included studies were highly heterogeneous, spanning RCTs, cohort studies, case series, and even prior reviews, with diverse patient populations, interventions, and outcome measures. This variability, coupled with the mix of study designs and quality, precluded any formal meta-analysis and necessitated a purely narrative synthesis of findings. Clinically, the evidence is difficult to generalise: some studies included different fracture presentations (e.g., occult or minimally displaced injuries alongside true Gartland I), and immobilisation protocols (cast vs splint type and duration of immobilization) varied widely across studies. Outcomes were reported inconsistently; for example, not all studies documented radiographic healing or adverse events, and most had short follow-up durations, limiting insight into long-term function and complication rates. Consequently, the review’s conclusions should be interpreted with caution given these methodological shortcomings and the limited, heterogeneous evidence base.

Future Research Priorities

Although the current evidence base is reassuring, there remain important gaps. Firstly, larger multicentre RCTs would be useful to directly compare removable casts and splints with sufficient power to assess rare complications. Large RCTs assessing longitudinal functional outcomes using validated patient-reported outcome measures would be useful to capture validated quality of life outcomes with adequate power. While novel materials (biobased, waterproof, and 3D printed) have shown superior patient satisfaction and comfort, analyses of their cost-effectiveness and incorporating clinic time, complications, and family costs (e.g., time off work) are required to assess how well these materials would fit into clinical practice. Furthermore, implementation studies would be useful to evaluate how best to introduce innovative immobilisation into routine paediatric orthopaedic practice. Finally, studies assessing equity considerations would be useful to understand whether innovative techniques could be accessible to all children or limited only to well-resourced settings.

Summary of Discussion

In summary, the literature confirms that Gartland I fractures are safe to manage with less restrictive immobilisation. The challenge is not stability but rather choosing the option that best balances comfort, convenience, safety, and resource efficiency. Posterior splints and removable casts provide the optimal mix of these attributes, while innovations promise further gains in satisfaction and daily function. To embed these findings into practice, clinicians must focus not only on device choice but also on quality of application, family education, and pragmatic follow-up strategies.

## Conclusions

For Gartland I supracondylar fractures, immobilisation choice does not affect fracture healing, which is consistently excellent. However, immobilisation does affect pain, function, satisfaction, and convenience. Evidence supports the use of posterior splints or removable long-arm casts as safe, effective, and family-friendly. Collar-and-cuff alone should generally be avoided. Novel materials offer added comfort and practicality, with potential system-level benefits. Clinicians should prioritise good application technique, patient or family education, and pragmatic follow-up. Future research should clarify cost-effectiveness and broader implementation of innovative materials.
